# Human Milk Oligosaccharides and Associations With Immune-Mediated Disease and Infection in Childhood: A Systematic Review

**DOI:** 10.3389/fped.2018.00091

**Published:** 2018-04-20

**Authors:** Alice M. Doherty, Caroline J. Lodge, Shyamali C. Dharmage, Xin Dai, Lars Bode, Adrian J. Lowe

**Affiliations:** ^1^Allergy and Lung Health Unit, Centre for Epidemiology and Biostatistics, Melbourne School of Population and Global Health, The University of Melbourne, Melbourne, VIC, Australia; ^2^Murdoch Children’s Research Institute, Melbourne, VIC, Australia; ^3^Division of Neonatology, Department of Pediatrics and Larsson-Rosenquist Foundation Mother-Milk-Infant Center of Research Excellence (LRF MoMI CoRE), University of California, San Diego, La Jolla, CA, United States; ^4^Division of Pediatric Gastroenterology and Nutrition, Department of Pediatrics and Larsson-Rosenquist Foundation Mother-Milk-Infant Center of Research Excellence (LRF MoMI CoRE), University of California, San Diego, La Jolla, CA, United States

**Keywords:** oligosaccharides, human milk, breastfeeding, infants, allergy and immunology, respiratory tract infections, diarrhea, HIV

## Abstract

Complex sugars found in breastmilk, human milk oligosaccharides (HMOs), may assist in early-life immune programming and prevention against infectious diseases. This study aimed to systematically review the associations between maternal levels of HMOs and development of immune-mediated or infectious diseases in the offspring. PubMed and EMBASE databases were searched (last search on 22 February 2018) according to a predetermined search strategy. Original studies published in English examining the effect of HMOs on immune-mediated and infectious disease were eligible for inclusion. Of 847 identified records, 10 articles from 6 original studies were included, with study quality ranging from low to high. Of three studies to examine allergic disease outcomes, one reported a protective effect against cow’s milk allergy (CMA) by 18 months of age associated with lower lacto-*N*-fucopentaose (LNFP) III concentrations (OR: 6.7, 95% CI 2.0–22). Another study found higher relative abundance of fucosyloligosaccharides was associated with reduced diarrhea incidence by 2 years, due to (i) stable toxin-*E. coli* infection (*p* = 0.04) and (ii) “all causes” (*p* = 0.042). Higher LNFP-II concentrations were associated with (i) reduced cases of gastroenteritis and respiratory tract infections at 6 weeks (*p* = 0.004, *p* = 0.010) and 12 weeks (*p* = 0.038, *p* = 0.038) and (ii) reduced HIV transmission (OR: 0.45; 95% CI: 0.21–0.97) and mortality risk among HIV-exposed, uninfected infants (HR: 0.33; 95% CI: 0.14–0.74) by 24 months. Due to heterogeneity of the outcomes reported, pooling of results was not possible. There was limited evidence that low concentrations of LNFP-III are associated with CMA and that higher fucosyloligosaccharide levels protect infants against infectious disease. Further research is needed.

## Introduction

Human milk contains a wide range of immunologically active components with the potential to protect against disease (1, 2). Research has emphasized the importance of human milk as an influential early-life exposure for the development of a healthy immune system; however, the mechanisms for this are still not clearly understood. Some studies have shown positive immunological effects and anti-infectious properties of human milk, particularly in the prevention of respiratory and gastrointestinal infections (3, 4). Other research suggests that breastfeeding influences the intestinal microbiome, which may in turn influence autoimmune and allergic disease development (4). The association between human milk and allergic disease is controversial, with numerous studies reporting inconsistent results (5). A possible explanation for the contradictory findings may lie in the diverse composition of bioactive factors present in human milk (6) and even when specific milk components are addressed, there may be significant differences in both quantity and variety between human milk from different mothers.

Human milk oligosaccharides (HMOs) are a key constituent of human milk. They are a structurally and biologically diverse group of complex indigestible sugars (7, 8). To date, more than 200 different oligosaccharides have been identified, varying in size from 3 to 22 monosaccharide units (9).

The most common HMOs are the neutral fucosylated and non-fucosylated oligosaccharides (10–13). The quantity and structure of these HMOs differs significantly among women and is dependent upon Secretor and Lewis blood group status (14, 15). Mutations in the fucosyltransferase 2 (FUT2) secretor gene results in human milk that is deficient in α1,2-linked fucosylated oligosaccharides (12).

Human milk oligosaccharides provide no direct nutritional value to the infant, and there is only minor absorption across the intestinal wall with approximately 1–5% detectable in serum and urine (8). It is proposed, instead, that HMOs have many different roles to play for the infant. They are preferred substrates for several species of gut bacteria and act as prebiotics, promoting the growth of beneficial intestinal flora and shaping the gut microbiome, thereby affecting immune responses (8, 16, 17). Short-chain fatty acids generated by the gut microbiome breaking down HMOs are critical for intestinal health. They further favor the growth of benign gut commensals along with providing nourishment for epithelial cells lining the intestine (18). HMOs also directly modulate host-epithelial responses, favoring reduced binding of pathogenic microbiota to the gut epithelium. Gut microbiota composition differs between formula-fed and breastfed infants, possibly due to the absence of HMOs in infant formula milk (19). There is also evidence that HMOs act as decoy receptors, inhibiting the binding of enteric pathogens to prevent infection and subsequent illness (20). Furthermore, HMOs provide a selective advantage for colonization by favorable bacteria, thereby inhibiting the growth of pathogenic species.

Despite substantial interest in this area, to date no systematic review has been undertaken to assess the effects of HMOs on disease prevention. This systematic review aims to identify and summarize the current evidence of the associations between HMOs and immune-mediated or infectious diseases in early childhood. Establishing a clear link between HMOs and disease outcomes may lead to intervention strategies.

## Methods

### Search Strategy

PubMed and EMBASE electronic databases were systematically searched (last search date 22 February 2018) for original studies examining the effect of HMOs on childhood immune-mediated and infectious disease outcomes. The search strategy included MeSH and free text terms for HMOs, allergic disease, immune-mediated disorders and clinical infections (see Table E1 in Supplementary Material). All original studies published in English were included. Papers that did not report original results, or outcome data of interest, were excluded.

Titles and abstracts of papers were screened by two authors (Alice M. Doherty and Xin Dai) for inclusion. Reference lists of primary articles and related reviews were checked to identify any other studies appropriate for inclusion. Studies assessed as eligible, potentially eligible or unclear, were retrieved in full text where available. Any uncertainty concerning inclusion of specific studies was resolved by discussion with a third author (Adrian J. Lowe). Outcomes of interest were the development of any immune-mediated diseases (allergic or autoimmune disorders) or clinical infections in childhood.

### Data Extraction

Study characteristics were extracted and tabulated from each of the included studies. The data extracted included the following: author’s name, date of publication, study design, location, population, exposure classification, outcome definitions, effect sizes, confounders and tests for potential effect modification, and potential sources of bias.

### Quality Assessment

The Newcastle-Ottawa scale was used to assess the quality of individual studies (21). The quality assessment was performed independently by two authors (AD, XD) to meet PRISMA guidelines. Each study was scored using a star (*) method to report the quality based on selection of sample, comparability, and the ascertainment of the exposure or outcome measures for case–control or cohort studies, respectively. Included studies were graded on total score: unsatisfactory = 0–3; low = 4–5; moderate = 6–7; and high = 8–9 (see Table E2 in Supplementary Material). Studies were not excluded based on quality assessment.

### Statistical Analysis

Where two or more papers reported the association between the same HMO and outcome, we pooled results using meta-analysis. The *I*^2^ statistic was used to document heterogeneity of study results, and random effect models were used where there was wide spread differences between studies (*I*^2^ > 80%).

## Results

**Figure 1:**
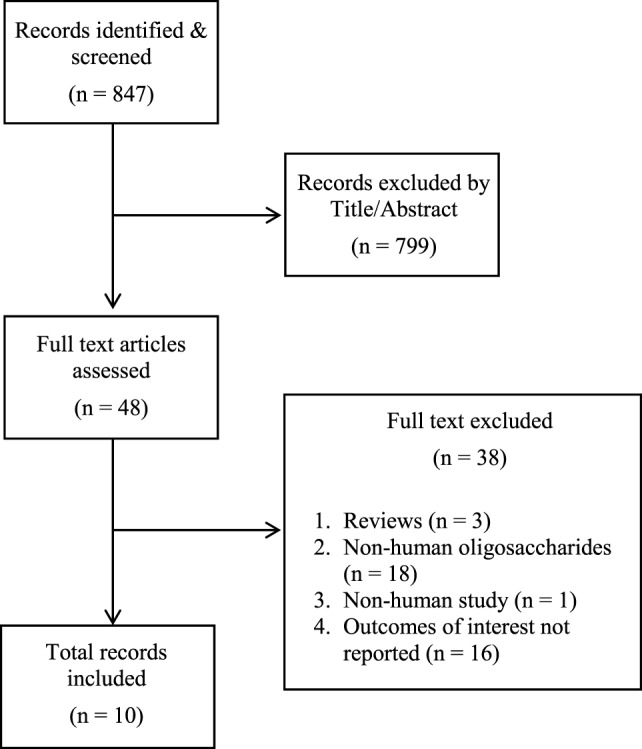
PRISMA flow diagram. Human milk oligosaccharides on immune-mediated and infectious disease outcomes.

The search identified 847 articles (see Figure 1). After title and abstract screening, 48 articles were selected for full-text assessment. In total, 10 records were included (see Table E3 in Supplementary Material for reasons for exclusion), from 6 original studies. Three articles reported on allergic disease outcomes (22–24), four articles on diarrheal disease outcomes, all from a single study (11, 25–27), one article on respiratory and gastrointestinal tract infections (28), and two articles on HIV outcomes from a single study (29, 30).

### Study Characteristics

Three prospective cohort studies assessed associations with allergic disease outcomes, all from Scandinavia, with sample sizes ranging from 20 (22) to 266 (23) mother–infant pairs (see Table 1). One study was population based (22), while the other two cohort studies sampled participants with parental allergic disease (23, 24). A prospective cohort of 93 mother–infant pairs conducted in Mexico City investigated associations between HMOs and infectious diarrhea (25). One prospective cohort study, of 73 participants, examined associations between HMOs and respiratory tract infections and gastroenteritis (28). The two publications examining associations with HIV outcomes were from one nested case–control study conducted in Lusaka, Zambia (29). Bode et al. reported HIV transmission from HIV-infected mothers to their exposed infants (29), and Kuhn et al. recorded mortality rates among HIV-exposed infants (30). Participants comprise 103 HIV infected and 143 HIV exposed but uninfected (HEU) children were randomly selected from an early weaning trial comprising of 958 HIV-infected mother–infant pairs (31).

**Table 1:** Characteristics of studies included examining the association between maternal levels of human milk oligosaccharides (HMOs) and allergic and infectious diseases.

Reference	Design	Location	Sample size	HMO exposure	Outcome
**Allergic disease**					
Sjögren et al. (22)	Prospective cohort	Sweden	20 mother–infant pairs	Concentration of 9 HMOs^a^ at 2–4 days of age	Any allergic disease^b^ at 18 months of age
Sprenger et al. (23)	Prospective cohort	Finland	266 mother–infant pairs	Levels of FUT2-dependent HMOs measured at mean 2.6 days of age	Any allergic disease^c^ and/or sensitization^d^ Eczema and/or sensitization At 2 and 5 years of age
Seppo et al. (24)	Prospective cohort	Finland	145 mother–infant pairs	Concentration of 19 HMOs^a^ at median 1.0–1.4 months of age	Cow’s milk allergy by 18 months of age
**Infectious disease**					
Newburg et al. (25)	Prospective cohort	Mexico	93 mother–infant pairs	Mean fucosyloligosaccharide ratios at 1–5 weeks postpartum	Diarrheal symptoms Clinical diarrhea At 2 years of age
Stepans et al. (28)	Prospective cohort	USA	73 mother–infant pairs	Levels of HMO *LNFP-II* at 2 weeks postpartum	Respiratory problems Gastrointestinal tract problems Ear infections At 2, 6, and 12 weeks of age
Bode et al. (29) and Kuhn et al. (30)	Nested case–control	Zambia	203 mother–infant pairs	Fucosyloligosaccharide concentration at 1 month postpartum	HIV infection (29) and mortality (30) at 24 months of age in children born to HIV-infected mothers

*HMO, human milk oligosaccharide; FUT2, fucosyltransferase 2; 2′FL, 2-linked fucosylated oligosaccharides; LNFP-II, lacto-N-fucopentaose II*.

*^a^Refer to Table E2 in Supplementary Material*.

*^b^Allergic disease defined as bronchial asthma, allergic rhinoconjunctivitis, atopic eczema, and food allergy*.

*^c^Allergic disease defined as asthma, allergic rhinitis, and eczema*.

*^d^Sensitization defined as any positive skin prick test*.

### HMO Assessment

Human milk oligosaccharides were quantified by high-performance liquid chromatography in all included studies. Sjögren and colleagues collected milk samples 2–4 days postpartum and reported exposure as median concentrations (nmol/mL) of nine common neutral oligosaccharides in colostrum (see Table E4 in Supplementary Material) (22). Sprenger et al. measured the presence or absence of FUT2-dependent oligosaccharides in mothers’ milk, collected at a mean postpartum day of 2.6; infants were classified as either FUT2-positive (had consumed levels of any FUT2-dependent HMOs) or as FUT2-negative (no FUT2-dependent HMOs) (23). Seppo et al. assessed concentration of 19 HMOs in milk samples at a median 1.0 month in mothers of non-cow’s milk allergy (CMA) infants and at median 1.4 months in mothers of CMA infants (24). An internal standard (Raffinose) was added to milk samples to allow for absolute quantification. Newburg et al. examined variations in fucosyloligosaccharides in human milk collected at 1–5 weeks postpartum (25). Exposure to HMOs was measured as the mean fucosylated oligosaccharide ratio: the concentrations of α1,2-linked fucosyloligosaccharides compared with oligosaccharides that contain only α1,3- and α1,4-linked fucose. Stepans et al. measured HMO exposure as levels of a major oligosaccharide, lacto-*N*-fucopentaose II (LNFP-II), at 2 weeks postpartum as a representative of HMO consumption (28). The HIV study defined the exposure of interest as HMO concentration, measured first as a continuous variable and second as a dichotomous variable with infants categorized as above or below the median HMO concentration (1.87 g/L) (29). Several different HMO groups were assessed. Human milk samples were collected at 1-month postpartum and were analyzed for HMO composition.

### Outcome Assessment

#### Allergic Disease

Sjögren et al. characterized children as “allergic” if clinical symptoms of allergic disease at 18 months of age were present and “non-allergic” if no clinical symptoms of allergic disease were apparent, along with a negative skin prick test (SPT) (22). Allergic disease was defined as bronchial asthma, allergic rhinoconjunctivitis, atopic eczema, and food allergy, although it was not clear if this was based on parent report or clinical examination. Sprenger measured the associations with any physician diagnosed allergic disease (food allergy, eczema, asthma, and allergic rhinitis) and/or IgE-associated disease (any allergic disease and sensitization as assessed by SPT) at 2 and 5 years of age (23). Seppo et al. measured outcomes of CMA by 18 months of age (24). Cases of CMA were confirmed by a positive oral food challenge at median 6 months of age.

#### Diarrhea

Outcomes reported were all diarrhea episodes due to stable toxin (ST)-*E. coli* infection and diarrhea as a result of all causes (25). Diarrheal episodes were determined by the study physician. All diarrheal episodes were assessed using a standardized scoring system (32, 33). ST-*E. coli* related diarrhea was tested in a laboratory according to previously published methods (34).

#### Respiratory and Gastrointestinal Tract Infections

Outcomes were cumulative occurrences of either (a) respiratory problems, consisting of upper respiratory infections (runny nose or cold), cough, or pneumonia; (b) gastrointestinal tract problems, which included vomiting, diarrhea, or colic; and (c) ear infections; by 2, 6, 12, and 24 weeks (28). Associations with ear infection outcomes were reported as not significant.

#### HIV

Bode et al. (29) reported the outcome measure as HIV transmission postpartum. The same study population was used to measure mortality in infants exposed to HIV infection during and after breastfeeding (30). HIV infection was established by heel-stick blood samples collected first at birth, then at 1 week of age, then monthly to 6 months of age, and subsequently every 3–24 months of age (29). HIV DNA was tested by polymerase chain reaction. Causes of deaths were ascertained *via* verbal autopsy and a review of medical records. Death after weaning was defined if breastfeeding had ceased independently of events before death (30).

### Study Quality

The included studies ranged from low to high quality (see Table E5 in Supplementary Material). Selection of participants was adequately reported for all included papers. The ascertainment of HMO exposure was based on laboratory assays for all included studies. Allergic disease outcomes were determined *via* parental reports in one study (22) and medical diagnosis for two studies (23, 24). While Newburg et al. reported physician confirmed diarrhea episodes (25), Stepans et al. used respiratory and gastrointestinal infections as reported by mothers (28). HIV status was confirmed *via* blood samples collected at several age intervals (29). Loss to follow-up was only reported in one study (28). Four of the six original studies considered possible bias as a result of confounding or effect modification (23, 24, 28, 29). One of the papers investigating allergic disease outcomes adjusted for potential confounders (siblings, delivery mode, gender, allergic parents, and gestational age) and tested for interactions (FUT2 status and delivery mode; and FUT2 status and siblings) (23), while another study adjusted for the age of the infant, maternal atopy, duration of lactation and Secretor status (24). Stepans et al. adjusted for breastfeeding behavior (28). Measures of association between HMOs and HIV transmission were adjusted for two potential confounders identified in the study, white blood cell count and human milk HIV RNA viral load at 1 month (29). Potential confounders and interaction terms for the association between HMOs and diarrheal diseases were reported as not significant; however, the authors did not discuss what covariates were tested (25). Sjögren et al. did not discuss confounding (22).

### Study Findings

#### Allergic Disease

One of the three allergic disease studies found evidence of an association between HMOs and allergic disease (22, 24). Sjögren reported a weak trend for higher total concentrations of neutral oligosaccharides in the breastmilk consumed by infants who developed allergic disease by 18 months (*p* = 0.12) (22) (see Table 2). Seppo observed that infants who consumed breastmilk with low lacto-*N*-fucopentaose (LNFP) III concentrations (<60 nM) had an increased likelihood of CMA compared with higher concentrations of LNFP-III (OR 6.7, 95% CI 2.0–22) (24). Seppo also noted that infants who received human milk with lower levels of LS-tetrasaccharide c, disialyllacto-*N*-tetraose (DSLNT) and 6′-sialyllactose were more likely to develop atopic dermatitis. Although Sprenger reported a significant interaction with mode of delivery (C-section or vaginal birth) (*p* = 0.016) (23), an adjusted regression model found no statistically significant association between oligosaccharide status and allergic disease, regardless of mode of delivery.

**Table 2:** Associations between HMO concentration and allergic disease outcomes.

Reference	Age (months)	Allergy status at 18 months		*n*	Conc. (g/L)^a^	*p*-Value
Sjögren et al. (22)	18	No allergy		11	7.53 (5.94–11.01)	0.12
		Allergy		9	9.88 (4.90–13.90)	

**Reference**	**Age (years)**	**Delivery mode**	**FUT2-positive (%)**	**FUT2-negative (%)**	**OR (95% CI)^b^**	***p*-Value**

Sprenger et al. (23)	2	Vaginal	32	27	1.3 (0.52–3.24)	0.658
		C-section	30	57	0.32 (0.06–1.6)	0.203

	5	Vaginal	52	42	1.47 (0.64–3.37)	0.406
		C-section	57	57	0.98 (0.20–4.94)	1.0

**Reference**	**Median (IQR) age in months**			***n***	**Median conc. (nM) LNFPII^a^**	***p*-Value**

Seppo et al. (24)	CMA		1.4 (0.7–2.8)	39	29	0.007
	No CMA		1.0 (0.12–1.9)	40	57	

	Atopic dermatitis		LSTc			0.019
			DSLNT			0.028
			6′SL			0.044

*FUT2, fucosyltransferase 2; CMA, cow’s milk allergy; LSTc, LS-tetrasaccharide c*.

*^a^Median concentration of HMOs*.

*^b^Unadjusted odds ratio*.

#### Diarrhea

It was found that infants with diarrhea due to ST-*E. coli* infection had a significantly lower mean fucosyloligosaccharide ratio than asymptomatically infected infants or uninfected infants (25) (see Table 3). In addition, lower fucosyloligosaccharide ratios were associated with more severe diarrheal disease due to any cause. Infants who developed moderate to severe diarrhea of any cause were fed with human milk that had lower fucosyloligosaccharides ratios than infants with no symptoms (25).

**Table 3:** Associations between human milk oligosaccharide concentration and diarrhea infection.

Reference	Cause of diarrhea infection		Mean fucosyloligosaccharide ratio^a^	SE	*p*-Value
Newburg et al. (25)	Stable toxin-*E. coli* infection	Infected, symptomatic	4.4	±0.8	
		Infected, asymptomatic	8.4	±1.0	0.04
		Uninfected	8.6	±1.1	0.04
	All causes	Moderate/severe symptoms	6.1	±0.9	
		No symptoms	10.5	±1.9	0.042

*^a^Mean fucosyloligosaccharide ratio refers to the concentrations of α1,2-linked fucosyloligosaccharides compared with oligosaccharides that contain only α1,3- and α1,4-linked fucose*.

#### Respiratory and Gastrointestinal Tract Infections

Higher levels of LNFP-II in colostrum were associated with reduced respiratory infections by 6 and 12 weeks, after controlling for breastfeeding behavior (see Table 4).

**Table 4:** The effect of HMO concentration on respiratory and gastrointestinal tract infections.

Reference	HMO	Time (weeks)	*N*	Odds ratio^a^	95% CI	*p*-Value
Stepans et al. (28)	*Respiratory tract infection*					

	LNFP-II	6	45	0.672	0.457, 0.989	0.01
		12	42	0.797	0.620, 1.026	0.04
		24	33	0.882	0.697, 1.115	0.28

	*Gastrointestinal tract infections*					

	LNFP-II	6	44	0.662	0.468, 0.935	0.004
		12	42	0.806	0.632, 1.029	0.04
		24	34	1.048	0.928, 1.182	0.41

*HMO, human milk oligosaccharide; LNFP-II, lacto-N-fucopentaose II*.

*^a^Associated change of odds due to a 1 μm LNFP-II change/mL milk*.

Increasing LNFP-II concentration was also associated with reduced gastrointestinal illness in infants at 6 and 12 weeks (26). No significant results were reported at 24 weeks postpartum.

#### HIV

There was a non-significant trend toward a reduction in HIV transmission risk postpartum (29). An association was found between higher total HMO concentration and reduced risk of HIV transmission after adjustment for maternal CD4 cell count and human milk HIV RNA viral load at 1 month (see Table 5). Assessment of individual oligosaccharides found non-3′-sialyllactose HMOs to reduce HIV transmission at concentrations above the median (OR 0.38; 95% CI: 0.17–0.82). No other significant reductions in HIV transmission were reported for the other oligosaccharides, instead higher concentrations of 3′-sialyllactose oligosaccharides were associated with an approximately 2-fold increased risk of transmission (adjusted OR: 2.21; 95% CI: 1.04–4.73) (29). For HEU infants, higher HMO concentrations were found to reduce mortality during, not after, breastfeeding for both 2-linked fucosylated as well as non-2-linked fucosylated oligosaccharides, following control for maternal CD4 cell count and human milk HIV RNA viral load at 1 month (30). No significant associations between HMOs and mortality were observed for HIV-infected children.

**Table 5:** Associations between HMO concentration and HIV transmission and mortality in children born to HIV-infected mothers.

HIV transmission			TR	NTR	OR (95% CI)	

Reference			*n*(%)	*n*(%)	Unadjusted	Adjusted^a^
Bode et al. (29)	Total log_10_ HMO conc.				0.43 (0.14–1.32)	0.31 (0.08–1.21)
	Median HMO conc. (g/L)	≥1.87	31 (41.9)	43 (58.1)	0.62 (0.34–1.15)	0.45 (0.21–0.97)
		<1.87	50 (53.8)	43 (46.2)		

**Mortality**					**Adjusted HR (95% CI)^b^**	

**Reference**	**Oligosaccharide**				**HIV infected**	**HEU**

Kuhn et al. (30)	Non-2′FL HMOs (LNFP-II/III + 3FL) > 200 mg/L				0.89 (0.38–2.08)	0.28 (0.13–0.67)
	2′FL + LNFP I > 550 mg/L				1.44 (0.64–3.21)	0.33 (0.14–0.74)
	LNT > 585 mg/L				1.43 (0.77–2.67)	0.58 (0.34–0.98)

*TR, HIV transmitting; NTR, HIV-non-transmitting; HEU, HIV-exposed uninfected; 2′FL, 2-linked fucosylated oligosaccharides; LNFP, lacto-N-fucopentaose; LNT, lacto-N-tetraose; 3FL, 3-fucosyllactose; HMO, human milk oligosaccharide*.

*^a^ORs adjusted for maternal CD4 cell count and log_10_ breast-milk HIV RNA viral load at 1 month*.

*^b^HRs adjusted for maternal CD4 cell count, mother symptomatic, maternal death, more than 2 other children aged <5 years in household, sex, and breastfeeding status*.

## Discussion

The six studies included in this systematic review have published 10 articles but provide only limited evidence that HMOs are associated with allergic and infectious diseases in early life. No studies were published on the associations between HMOs and other immune-mediated conditions. In terms of allergic disease, only one study showed that LNFP III was associated with increased risk of CMA. For infectious disease, the evidence was stronger although still limited with one study reporting that increased maternal levels of fucosyloligosaccharides were associated with reduced risk of diarrhea up to 2 years of age, as well as respiratory and gastrointestinal tract infections at 6 and 12 weeks of age. Evidence for an association between high HMO concentration and HIV outcomes was reported in two studies. In infants with a HIV positive mother, HMOs above the median concentration (≥1.87 g/L) had reduced risk of HIV infection. In addition, high concentrations of HMOs during breastfeeding were associated with a lower mortality rate for HIV-exposed, uninfected (HEU) infants but not for HIV-exposed infected infants.

Despite the recent interest and discussion surrounding breastmilk oligosaccharides and their potential impact on disease outcomes, very little original research has been conducted in this field. Furthermore, there are a number of important limitations with the available evidence, which prohibit strong conclusions being made at this time. Where two or more studies examined associations with similar outcomes, different measures of association were used (odds ratios versus mean differences in maternal HMO between affected and unaffected infants), preventing any pooling of the results in a meta-analysis. Most of the included studies measured disease outcomes from very small sample sizes (between 20 and 266 mother–infant pairs) both affecting the precision of the effect estimates and limiting the statistical power to detect important associations.

There were disparate HMO exposure classifications between the studies, making it difficult to compare the results of each study. Sprenger et al. measured exposure to HMOs as presence or absence of FUT2-dependent oligosaccharides (23). Using this dichotomy assumes that infants who consume high concentrations of FUT2-dependent oligosaccharides are just as likely to develop allergic disease as those exposed to low concentrations. Newburg et al. defined exposure to HMOs in terms of fucosylated oligosaccharide ratios (concentrations of α1,2-linked fucosyloligosaccharides compared with oligosaccharides that contain only α1,3- and α1,4-linked fucose), thus grouping a range of HMOs (25), whereas the remaining four studies measured associations with several specific common HMOs (22, 24, 28, 29). Furthermore, most of the techniques used to measure levels of HMO are unable to quantify absolute levels of HMO. Only three papers discussed using an internal standard in the HMO quantification process (23, 24, 29).

With over 200 structurally unique HMOs, it is possible that other important oligosaccharides not considered in the six studies may have important biological effects. It remains possible that the included studies have not measured the important HMO for each of the assessed outcomes. However, with so many different forms of HMOs, testing associations for each HMO will result in multiple comparisons, leading to spurious associations. As this area is novel, such exploratory “hypothesis generating” studies are still needed, but, care should be taken not to over-interpret any one finding in the absence of replication across cohorts.

Potential confounding was accounted for differently in each study. Four of the six original studies adjusted for potential confounders (see Figures E1–E3 in Supplementary Material) (23, 24, 28, 29). The remaining articles failed to acknowledge possible confounding (22) or stated confounding was not significant, with no description of how this was confirmed (25). Two of the allergic disease studies adjusted for a range of potential confounders in their analysis including siblings, gender, allergic parents/maternal atopy, and gestational age, which largely appears appropriate (23, 24). Bode et al. noted that associations between advanced maternal HIV infection, including low maternal CD4 counts and viral load, and higher HEU mortality were confined to breastfed children, thus CD4 counts and viral load were controlled for in the regression model (29). Stepans et al. controlled for breastfeeding behavior in the analysis; defined as the proportion of days breastfed (28), but it is unlikely that the duration of breastfeeding would confound the relationship between HMO consumption and disease outcomes as maternal levels of HMOs were measured for all women at one fixed time window—2 weeks after recruitment. As all of the available evidence is derived from observational studies (cohort and nested case–control), it is subject to potential unmeasured confounding factors.

It is possible that a range of factors modify the effects of HMOs, and this has been examined in some of the included studies. Delivery mode was reported to be a significant modifier of the association between the presence of FUT2-dependent oligosaccharides and allergic disease development, although the strata-specific effects were quite weak (23). It is possible that breastfeeding duration is an effect modifier; longer breastfeeding duration may result in a larger amount of HMOs consumed by the infant, thereby affecting outcomes. Similarly, total volume of breastmilk consumed may also vary between infants and could modify these associations. This possibility has not been examined previously.

Follow-up was complete for five of the six studies (22–25, 29). However, it is not clear whether the authors only reported results on participants with outcome data. Stepans reported that only 34 participants from the original sample of 73 (46%) remained after 24 weeks, leading to a high risk of attrition bias.

The generalizability of results may be affected by population homogeneity in the regions in which participants were recruited. As the proportion of women with FUT2 gene mutations varies in different ethnic populations, lack of genetic diversity among study participants may result in associations that are unique to that specific population. For example, in the study by Newburg et al., all participants secreted fucosylated HMOs (no mutations in the FUT2 gene in the Mexican population). This meant that there was less variation in the levels of FUT2-dependent oligosaccharides and hence higher fucosyloligosaccharide ratios were reported than would potentially be found in other ethnic populations. Therefore, these results cannot be directly translated to European populations where there is a higher proportion of non-secretors (35, 36). An additional limitation in these studies is that while the Secretor status of the mother is often known, based on expressed breastmilk HMOs, the status of the infant has not been measured. Secretor status of the child may also modify the associations between HMO and clinical outcomes. However, infant Secretor status is difficult to measure.

This review has a number of strengths and limitations. We prospectively registered the review, searched multiple databases, had duplicate study selection, and listed reasons for excluding studies and quality assessed the included papers. While only published works were included in the predetermined search strategy, additional sources in the form of conversations with colleagues were used to identify possible unpublished manuscripts. Publication bias may have influenced the results of this review, although this seems unlikely given the preponderance of negative associations that have been reported to date. While our search strategy allowed for their inclusion, none of the published papers reported outcomes beyond infancy or reported autoimmune disease outcomes, which are areas for future research.

## Conclusion

We identified limited evidence to support a possible role for HMOs to influence cows’ milk allergy, diarrheal diseases, respiratory, gastrointestinal tract infections, and HIV infection in the infant in early life. Despite these positive findings, the evidence base is very limited and has numerous issues, with varying quality of the included studies from low to high. Further research into this area is needed, using larger observational studies with appropriate measures of outcomes and exposures and better control for confounding. Future research would benefit from considering multiple HMO exposures, which could be grouped appropriately, either by similar biological effects or by patterns of associations. Improved understanding of the complex chemical structures of oligosaccharides in milk may potentially allow for the design of intervention studies, to increase exposure to specific HMOs, which may reduce the burden of these conditions.

## Author Contributions

AL and LB conceived the work. AD developed the search strategy and AD, AL, and XD systematically assessed studies for inclusion. AD developed the first draft manuscript, with input from AL, and all authors drafted and revised the manuscript and approved the final submitted version.

## Conflict of Interest Statement

The authors declare that the research was conducted in the absence of any commercial or financial relationships that could be construed as a potential conflict of interest.
