# Supplementary material for: Human Milk Oligosaccharides and Associations With Immune-Mediated Disease and Infection in Childhood: A Systematic Review
Source: Front Pediatr. 2018 Apr 20;6:91. doi: 10.3389/fped.2018.00091 (PMC5920034; doi:10.3389/fped.2018.00091)
Supplement: Supplementary file 1 [file Table_1.DOCX]

**Table E1.** Search Strategy for human milk oligosaccharides and immune-mediated and infectious disease outcomes in databases PubMed and EMBASE

| PUBMED | EMBASE |
| --- | --- |
| #1 Oligosaccharides [all fields]  #2 “Carbohydrates”[MeSH terms]  #3 Saccharides [All fields]  #4 Glycans [All fields]  #5 #1 OR #2 OR #3 OR #4 | #1 Oligosaccharides/  #2 Carbohydrates/  #3 Saccharides  #4 Glycans  #5 1 OR 2 OR 3 OR 4 |
| #6 "Milk, Human"[MeSH terms]  #7 "Breast Feeding"[MeSH terms]  #8 Breast[All Fields] AND Feed*[All Fields]  #9 Breast-fe*[All Fields]  #10 Infant fe* [All Fields]  #11 Infant nutrition* [All Fields]  #12 Colostrum [All fields]  #13 #5 OR #6 OR #7 OR #8 OR #9 OR #10 OR #11 OR #12 | #6 exp breast feeding/  #7 exp breast milk/  #8 Breast AND Feed*  #9 Breast-fe*  #10 Infant fe*  #11 Infant nutrition*  #12 Colostrum  #13 6 OR 7 OR 8 OR 9 OR 10 OR 11 OR 12 |
| #14 “Asthma”[MeSH terms]  #15 "Respiratory Sounds"[MeSH terms]  #16 Wheez*[All Fields]  #17 "Eczema"[MeSH terms]  #18 "Dermatitis, Atopic"[MeSH terms]  #19 atopic eczema* [All Fields]  #20 "Rhinitis, Allergic, Seasonal"[MeSH terms]  #21 "Rhinitis, Allergic, Perennial"[MeSH terms]  #22 Allergic rhinitis [All fields]  #23 Hay fever [All fields]  #24 Pollinosis [All Fields]  #25 “Conjunctivitis, Allergic”[MeSH terms]  #26 Rhinoconjunctivitis [All Fields]  #27 “Allergy and Immunology”[MeSH terms]  #28 Allergy [all fields]  #29 “Hypersensitivity”[MeSH terms]  #30 "Food Hypersensitivity"[MeSH terms]  #31 Food allerg*[All Fields]  #32 Food hypersensit*[All Fields]  #33 #14 OR #15 OR #16 OR #17 OR #18 OR #19 OR #20 OR #21 OR #22 OR #23 OR #24 OR #25 OR #26 OR #27 OR #28 OR #29 OR #30 OR #31 OR #32 | #14 exp asthma/  #15 exp wheezing/  #16 wheez*  #17 exp eczema/  #18 exp atopic dermatitis/  #19 atopic eczema*  #20 exp allergic rhinitis/  #21 allergic rhinitis  #22 hay fever  #23 pollinosis  #24 conjunctivitis  #25 rhinoconjunctivitis  #26 exp Allergy/  #27 exp Immunology/  #28 allergy  #29 exp Hypersensitivity/  #30 exp food allergy/  #31 food allerg*  #32 food hypersensit*  #33 14 OR 15 OR 16 OR 17 OR 18 OR 19 OR 20 OR 21 OR 22 OR 23 OR 24 OR 25 OR 26 OR 27 OR 28 OR 29 OR 30 OR 31 OR 32 |
| #34 “Autoimmune Diseases”[MeSH terms]  #35 “Diabetes Mellitus, Type 1”[MeSH terms]  #36 Diabetes [All Fields]  #37 Diabetic [All Fields]  #38 “Graves Disease”[MeSH terms]  #39 Basedow* disease [All Fields]  #40 exophthalmic goiter [All Fields]  #41 “Thyroiditis, Autoimmune”[MeSH terms]  #42 “Arthritis, Juvenile”[MeSH terms]  #43 “Arthritis, Rheumatoid”[MeSH terms]  #44 “Hashimoto Disease” [MeSH terms]  #45 “Inflammatory Bowel Diseases”[MeSH terms]  #46 “Crohn Disease”[MeSH terms]  #47 “Colitis, Ulcerative”[MeSH terms]  #48 “Celiac Disease”[MeSH terms]  #49 Coeliac [All Fields]  #50 “Prurigo”[MeSH terms]  #51 “Psoriasis”[MeSH terms]  #52 “Vitiligo”[MeSH terms]  #53 #34 OR #35 OR #36 #37 OR #38 OR #39 OR #40 OR #41 OR #42 OR #43 OR #44 OR #45 OR #46 OR #47 OR #48 OR #49 OR #50 OR #52 | #34 exp autoimmune diseases/  #35 (Diabetes Mellitus Type 1)  #36 diabetes  #37 diabetic  #38 (Graves adj3 disease)  #39 Basedow  #40 exophthalmic goiter.  #41 thyroiditis  #42 (arthritis adj3 juvenile)  #43 (arthritis adj3 rheumatoid)  #44 (Hashimoto adj3 disease)  #45 (Ulcerative adj3 colitis)  #46 (Inflammatory Bowel Diseases)  #47 Crohn Disease/  #48 Celiac Disease/  #49 coeliac  #50 Prurigo/  #51 Psoriasis/  #52 Vitiligo/  #53 34 OR 35 OR 36 OR 37 OR 38 OR 39 OR 40 OR 41 OR 42 OR 43 OR 44 OR 45 OR 46 OR 47 OR 48 OR 49 OR 50 OR 51 OR 52 |
| #54 “Bacterial infections and mycoses”[MeSH terms] | #54 Bacterial infections |
| #55 “Respiratory tract diseases”[MeSH terms] | #55 diarrhea |
| #56 infections[All Fields] | #56 diarrhoea |
| #57 infectious disease[All Fields] | #57 Respiratory tract infections |
| #58 #54 OR #55 OR #56 OR #57 | #58 infections |
|  | #59 infectious disease |
|  | #60 54 OR 55 OR 56 OR 57 OR 58 OR 59 |
| #59 #5 AND #13 AND (#33 OR #53 OR #58) | #61 5 AND 13 AND (33 OR 53 OR 60) |
| Total: 697 | Total: 306 |
