# Supplementary material for: Human Milk Oligosaccharides and Associations With Immune-Mediated Disease and Infection in Childhood: A Systematic Review
Source: Front Pediatr. 2018 Apr 20;6:91. doi: 10.3389/fped.2018.00091 (PMC5920034; doi:10.3389/fped.2018.00091)
Supplement: Supplementary file 2 [file Table_2.DOCX]

**Table E2.** Application of Newcastle-Ottawa grading system criteria within this review.

| Criteria |  | |  | Score |
| --- | --- | --- | --- | --- |
| Cohort studies | | | | |
| Selection | *Representativeness of exposed* | Not documented or selected group e.g. nurses, volunteers | | 0 |
|  |  | Truly, or somewhat, representative of average pregnant woman in the community | | 1 |
|  | *Selection of non-exposed cohort* | Not documented or drawn from different source to exposed cohort | | 0 |
|  |  | Drawn from same community as exposed cohort | | 1 |
|  | *Ascertainment of exposure* | Not documented or written parental report | | 0 |
|  |  | Laboratory record | | 1 |
|  | *Outcome not present at start of study* | No | | 0 |
|  |  | Yes – for participants pregnant at commencement of study | | 1 |
| Comparability | | Not documented | | 0 |
|  |  | Adjusted for important factor: family history | | 1 |
|  |  | Adjusted for additional factor(s): older siblings, gestational age, secretor status, gender | | 2 |
| Outcome | *Assessment of outcome* | Not documented or parental report | | 0 |
|  |  | Study physician-diagnosed outcome or medical record | | 1 |
|  | *Adequate follow up period* | No | | 0 |
|  |  | Yes | | 1 |
|  | *Adequacy of follow up of cohort* | Not documented or <70% follow-up | | 0 |
|  |  | > 70 follow-up | | 1 |

| **Case-control studies** | |  |  |
| --- | --- | --- | --- |
| Selection | *Case definition adequate* | No description | 0 |
|  |  | Yes, record linkage or based on parental reports | 0 |
|  |  | Yes, primary records e.g. medical records | 1 |
|  | *Representativeness of cases* | Not documented or potential for selection bias | 0 |
|  |  | All eligible cases with outcome of interest over defined period of time, all cases  in defined area/hospital/clinic or a random sample of those cases | 1 |
|  | *Selection of controls* | Not documented or hospital controls | 0 |
|  |  | Community controls | 1 |
|  | *Definition of controls* | No description of source | 0 |
|  |  | No history of disease | 1 |
| Comparability | | Not documented | 0 |
|  |  | Adjusted for important factor: family history | 1 |
|  |  | Adjusted for additional factor(s): older siblings, gestational age, secretor status, gender | 2 |
| Exposure | *Ascertainment of exposure* | Not documented or written parental report | 0 |
|  |  | Laboratory record | 1 |
|  | *Same ascertainment method* | No | 0 |
|  |  | Yes | 1 |
|  | *Same nonresponse rate* | Not documented or non-respondents described but response rate different | 0 |
|  |  | Same rate for both groups | 1 |

| Score | Quality of Study |
| --- | --- |
| 0 – 3 | Unsatisfactory |
| 4 – 5 | Low |
| 6 – 7 | Moderate |
| 8 – 9 | High |
