# Supplementary material for: Human Milk Oligosaccharides and Associations With Immune-Mediated Disease and Infection in Childhood: A Systematic Review
Source: Front Pediatr. 2018 Apr 20;6:91. doi: 10.3389/fped.2018.00091 (PMC5920034; doi:10.3389/fped.2018.00091)
Supplement: Supplementary file 3 [file Table_3.DOCX]

**Table E3.** List of excluded articles

| Excluded article | | Reason* |
| --- | --- | --- |
| 1 | Lenoir-Wijnkoop, I., et al. Nutrition economic impact of a specific mixture of prebiotic oligosaccharides in primary prevention of atopic dermatitis. 30th Congress of the European Academy of Allergy and Clinical Immunology, Istanbul. Allergy 2001;66:615. | X1, X2 |
| 2 | Hoekstra, J.H., et al. Oral rehydration solution containing a mixture of non-digestible carbohydrates in the treatment of acute diarrhea: a multicenter randomized placebo controlled study on behalf of the ESPGHAN working group on intestinal infections. Journal of pediatric gastroenterology and nutrition 2004;39(3):239-245. | X2 |
| 3 | Morrow, A.L., et al. Human-milk glycans that inhibit pathogen binding protect breast-feeding infants against infectious diarrhea. J Nutr 2005;135(5):1304-7. | X2, X4 |
| 4 | Newburg, D.S., et al. Innate protection conferred by fucosylated oligosaccharides of human milk against diarrhea in breastfed infants. Glycobiology 2004;14(3):253-263. | X3 |
| 5 | Newburg, D.S., et al. Human milk glycans protect infants against enteric pathogens. Annual Review of Nutrition 2005;25:37-58. | X1, X2 |
| 6 | Coppa, G.V., et al. Human milk oligosaccharides inhibit the adhesion to Caco-2 cells of diarrheal pathogens: Escherichia coli, Vibrio cholerae, and Salmonella fyris. Pediatr Res 2006;59(3):377-82. | X1 |
| 7 | Moro, G., et al. A mixture of prebiotic oligosaccharides reduces the incidence of atopic dermatitis during the first six months of age. Archives of Disease in Childhood 2006;91(10):814-819. | X2 |
| 8 | Arslanoglu, S., et al. Early supplementation of prebiotic oligosaccharides protects formula-fed infants against infections during the first 6 months of life. J Nutr 2007;137(11):2420-4. | X2 |
| 9 | Kukkonen, K., et al. Probiotics and prebiotic galacto-oligosaccharides in the prevention of allergic diseases: A randomized, double-blind, placebo-controlled trial. Journal of Allergy and Clinical Immunology 2007;119(1):192-198. | X2 |
| 10 | Arslanoglu, S., et al. Early dietary intervention with a mixture of prebiotic oligosaccharides reduces the incidence of allergic manifestations and infections during the first two years of life. J Nutr 2008;138(6):1091-5. | X2 |
| 11 | Westerbeek, E.A., et al. Design of a randomised controlled trial on immune effects of acidic and neutral oligosaccharides in the nutrition of preterm infants: carrot study. BMC Pediatr 2008;8:46. | X1, X2 |
| 12 | Bruzzese, E., et al. A formula containing galacto- and fructo-oligosaccharides prevents intestinal and extra-intestinal infections: An observational study. Clinical Nutrition 2009;28(2):156-161 | X2 |
| 13 | Hong, P., et al. Human milk oligosaccharides reduce HIV-1-gp120 binding to dendritic cell-specific ICAM3-grabbing non-integrin (DC-SIGN). Br J Nutr 2009;101(4):482-6. | X1 |
| 14 | Shibata R., et al. Clinical effects of prebiotics with kestose, a fructo-oligosaccharide, on atopic dermatitis in infants. 28th Congress of the European Academy of Allergy and Clinical Immunology Abstract Book, Warszawa. Allergy 2009;64:447. | X2 |
| 15 | Van Hoffen, E., et al., A specific mixture of short-chain galacto-oligosaccharides and long-chain fructo-oligosaccharides induces a beneficial immunoglobulin profile in infants at high risk for allergy. Allergy 2009;64(3):484-487. | X1, X2 |
| 16 | Eiwegger, T., et al. Prebiotic oligosaccharides: in vitro evidence for gastrointestinal epithelial transfer and immunomodulatory properties. Pediatr Allergy Immunol 2010;21(8):1179-88. | X1, X2 |
| 17 | Modi, N., et al. A randomized, double-blind, controlled trial of the effect of prebiotic oligosaccharides on enteral tolerance in preterm infants. Pediatr Res 2010; 68(5):440-5. | X1, X2 |
| 18 | Vivatvakin, B., et al. Effect of a whey-predominant starter formula containing LCPUFAs and oligosaccharides (FOS/GOS) on gastrointestinal comfort in infants. Asia Pac J Clin Nutr, 2010. 19(4): p. 473-80. | X1, X2 |
| 19 | Westerbeek, E.A., et al. Neutral and acidic oligosaccharides in preterm infants: a randomized, double-blind, placebo-controlled trial. Am J Clin Nutr 2010;91(3):679-86. | X1, X2 |
| 20 | Arslanoglu, S., et al. Neutral prebiotic oligosaccharide supplementation early in life and allergy associated symptoms later on: A 5-year follow-up. European Society for Paediatric Gastroenterology, Hepatology, and Nutrition Annual Meeting 2011, Sorrento. Journal of Pediatric Gastroenterology and Nutrition 2011;52:E66-E67. | X2 |
| 21 | De Kivit S., et al. Dietary non-digestible oligosaccharide-induced galectin-9 correlates with protection against allergic symptoms. Pharma-Nutrition Symposium 2011, Amsterdam. European Journal of Pharmacology 2011;668:e14 | X2 |
| 22 | Nasir M. et al. Bifidogenicity of galacto-oligosaccharides in diarrhea management of acute malnourished infants and young children. 70th Anniversary of the Nutrition Society Winter Meeting 2011, London. Proceedings of the Nutrition Society 2011;70(6):E344 | X1, X2 |
| 23 | Niele, N., et al. Effect of enteral supplementation of neutral and acidic oligosaccharides in preterm infants on allergic and infectious diseases during the first year of life. European Society for Paediatric Gastroenterology, Hepatology, and Nutrition Annual Meeting 2011, Sorrento. Journal of Pediatric Gastroenterology and Nutrition 2011;52:E115-E116 | X2 |
| 24 | Schouten, B., et al. Non-digestible oligosaccharides reduce immunoglobulin free light-chain concentrations in infants at risk for allergy. Pediatric Allergy and Immunology 2011;22(5):537-542. | X2 |
| 25 | Westerbeek, E.A., et al. Effect of neutral and acidic oligosaccharides on fecal IL-8 and fecal calprotectin in preterm infants. Pediatr Res 2011;69(3):255-8. | X1, X2 |
| 26 | Moro, G.E., et al. Early neutral prebiotic oligosaccharide supplementation reduces the incidence of some allergic manifestations in the first 5 years of life. J Biol Regul Homeost Agents. 2012;26(3 Suppl):49-59 | X2 |
| 27 | Osborn, D.A. and J.K. Sinn. Prebiotics in infants for prevention of allergic disease and food hypersensitivity. 17th Congress of the Federation of Asian and Oceania Perinatal Societies, FAOPS and the 16th Annual Congress of the Perinatal Society of Australia and New Zealand, Sydney. Journal of Paediatrics and Child Health 2012;48:25. | X2 |
| 28 | Ivakhnenko, O.S. and S.L. Nyankovskyy. Effect of the specific infant formula mixture of oligosaccharides on local immunity and development of allergic and infectious disease in young children: Randomized study. Pediatria Polska 2013; 88(5):398-404. | X2 |
| 29 | Weichert, S., et al. Bioengineered 2'-fucosyllactose and 3-fucosyllactose inhibit the adhesion of Pseudomonas aeruginosa and enteric pathogens to human intestinal and respiratory cell lines. Nutr Res 2013;33(10):831-8. | X1, X2 |
| 30 | Armanian, A.M., et al. The Effect of Neutral Oligosaccharides on Reducing the Incidence of Necrotizing Enterocolitis in Preterm infants: A Randomized Clinical Trial. Int J Prev Med 2014;5(11):1387-95. | X2 |
| 31 | Lehmann S. et al. Additive effect of non-digestible oligosaccharides on lactic acid bacteria induced secretion of anti-inflammatory IL-10 by human monocyte derived dendritic cells. 41st Annual Meeting of the Arbeitsgemeinschaft Dermatologische Forschung, ADF 2014, Cologne. Experimental Dermatology 2014; 23(3):e31. | X1, X2 |
| 32 | Lin, A., et al. Human milk oligosaccharides protect bladder epithelial cells against uropathogenic E. Coli and Streptococcus agalactiae infections. Experimental Biology 2014, EB San Diego. FASEB Journal 2014;28 (1 SUPPL. 1). | X1 |
| 33 | Lin, A.E., et al. Human milk oligosaccharides protect bladder epithelial cells against uropathogenic Escherichia coli invasion and cytotoxicity. J Infect Dis 2014;209(3):389-98. | X1 |
| 34 | Van Niekerk, E., et al. Human milk oligosaccharides differ between HIV-infected and HIV-uninfected mothers and are related to necrotizing enterocolitis incidence in their preterm very-low-birth-weight infants. J Nutr 2014;144(8):1227-33. | X1 |
| 35 | Gruber, C., et al. Prevention of early atopic dermatitis among low-atopy-risk infants by immunoactive prebiotics is not sustained after the first year of life. 34th Congress of the European Academy of Allergy and Clinical Immunology, Barcelona. Allergy 2015;70:286-287. | X2 |
| 36 | Olivares, M., et al. Human milk composition differs in healthy mothers and mothers with celiac disease. Eur J Nutr 2015;54(1):119-28. | X1 |
| 37 | He, Y., et al. The human milk oligosaccharide 2'-fucosyllactose modulates CD14 expression in human enterocytes, thereby attenuating LPS-induced inflammation. Gut 2016;65(1):33-46. | X1 |
| 38 | Newburg, D.S., et al. Human Milk Oligosaccharides and Synthetic Galactosyloligosaccharides Contain 3'-, 4-, and 6'-Galactosyllactose and Attenuate Inflammation in Human T84, NCM-460, and H4 Cells and Intestinal Tissue Ex Vivo. J Nutr 2016;146(2):358-67. | X1, X2 |

X1 – Did not report relevant outcomes; X2 - Exposure was to oligosaccharides that ‘mimic’ human milk oligosaccharides but are synthesised and not found in human breast milk, e.g. galacto-oligosaccharides and fructo-oligosaccharides; X3 – Duplicate of an included study; X4 – Not an original study, e.g. review
