# Supplementary material for: Human Milk Oligosaccharides and Associations With Immune-Mediated Disease and Infection in Childhood: A Systematic Review
Source: Front Pediatr. 2018 Apr 20;6:91. doi: 10.3389/fped.2018.00091 (PMC5920034; doi:10.3389/fped.2018.00091)
Supplement: Supplementary file 4 [file Table_4.DOCX]

**Table E4.** Human milk oligosaccharide classification

| **Abbreviation** | **Name** | **Study*** |
| --- | --- | --- |
| 2'FL | 2'-fucosyllactose | 1, 2 |
| 3FL | 3-fucosyllactose | 1, 2 |
| LNnT | Lacto-N-neotetraose | 1, 2 |
| 3'SL | 3'-sialyllactose | 2 |
| DFLac | Difucosyllactose | 1, 2 |
| 6'SL | 6'-sialyllactose | 2 |
| LNT | Lacto-N-tetraose | 1, 2 |
| LNFP I | Lacto-N-fucopentaose I | 1, 2 |
| LNFP II | Lacto-N-fucopentaose II | 1, 2 |
| LNFP III | Lacto-N-fucopentaose III | 1, 2 |
| LSTb | LS-tetrasaccharide b | 2 |
| LSTc | LS-tetrasaccharide c | 2 |
| DFLNT | Difucosyllacto-N-tetraose | 2 |
| LNH | Lacto-N-hexaose | 2 |
| DSLNT | Disialyllacto-N-tetraose | 2 |
| FLNH | Fucosyllacto-N-hexaose | 2 |
| DFLNH | Difucosyllacto-N-hexaose | 2 |
| FDSLNH | Fucosyl-disialyllacto-N-hexaose | 2 |
| DSLNH | Disialyllacto-N-hexaose | 2 |
| LNDFH I | lacto-N-difucohexaose I | 1 |

* where ‘1’ is the Sjögren et al study [21] and ‘2’ the Seppo et al study [24]
