# Supplementary material for: Human Milk Oligosaccharides and Associations With Immune-Mediated Disease and Infection in Childhood: A Systematic Review
Source: Front Pediatr. 2018 Apr 20;6:91. doi: 10.3389/fped.2018.00091 (PMC5920034; doi:10.3389/fped.2018.00091)
Supplement: Supplementary file 5 [file Table_5.DOCX]

**Table E5** Newcastle-Ottawa assessment^1^ of quality of evidence

| **Cohort studies** | **Allergic disease** | | | | | **Diarrhoea** | **RTI & GI** |
| --- | --- | --- | --- | --- | --- | --- | --- |
|  | Sjögren (2007) [22] | Sprenger (2016) [23] | | Seppo (2016) [24] | | Newburg (2004) [25] | Stepans (2006) [28] |
| 1. *Representativeness of exposed* | * | * | | * | | - | * |
| 1. *Selection of non-exposed* | * | * | | * | | * | * |
| 1. *Ascertainment of exposure* | * | * | | * | | * | * |
| 1. *Outcome not present at start of study* | * | * | | * | | * | * |
| 1. *Comparability* | - | ** | | ** | | - | - |
| 1. *Assessment of outcome* | - | * | | * | | * | - |
| 1. *Adequate follow up period* | * | * | | * | | * | * |
| 1. *Adequacy of follow up of cohort* | * | * | | * | | * | - |
| *Total^2^* | 6 | 9 | | 9 | | 6 | 5 |
| **Case-control studies** | **HIV** | | | |  | | |
|  | Bode  (2012) [29] | | Kuhn  (2015) [30] | |  | | |
| 1. *Case definition adequate* | * | | * | |  | | |
| 1. *Representativeness of cases* | * | | * | |  | | |
| 1. *Selection of controls* | * | | * | |  | | |
| 1. *Definition of controls* | * | | * | |  | | |
| 1. *Comparability* | * | | * | |  | | |
| 1. *Ascertainment of exposure* | * | | * | |  | | |
| 1. *Same ascertainment method* | * | | * | |  | | |
| 1. *Same nonresponse rate* | * | | * | |  | | |
| *Total* | 8 | | 8 | |  | | |

RTI, respiratory tract infection; GI, gastrointestinal problems

^1^Based on the Newcastle-Ottawa grading system [21]; ^2^Score quality rating: unsatisfactory = 0–3; low = 4–5; moderate = 6–7; high = 8–9
