# Supplementary material for: Human Milk Oligosaccharides and Associations With Immune-Mediated Disease and Infection in Childhood: A Systematic Review
Source: Front Pediatr. 2018 Apr 20;6:91. doi: 10.3389/fped.2018.00091 (PMC5920034; doi:10.3389/fped.2018.00091)
Supplement: Supplementary file 7 [file Image_2.PDF]

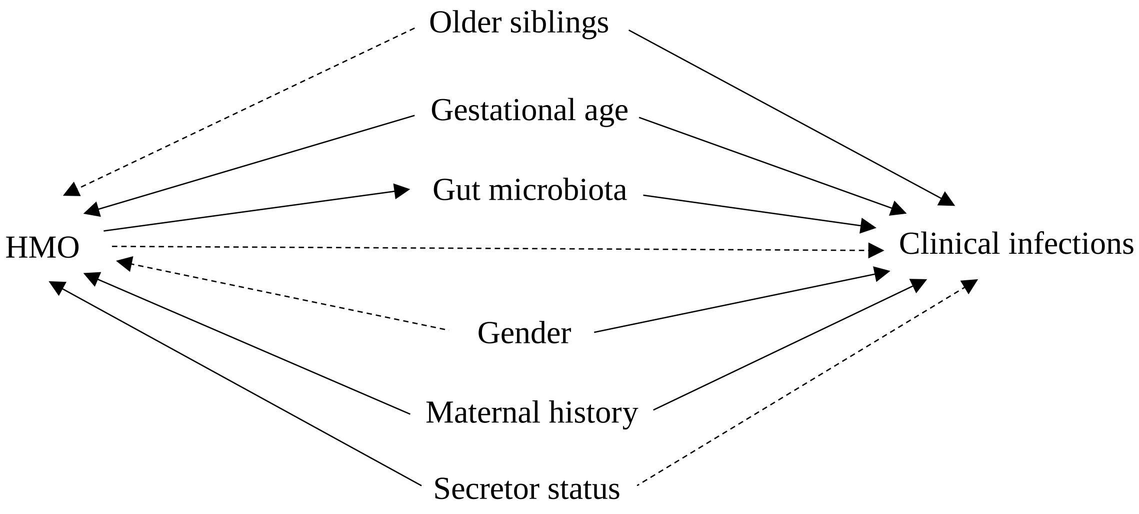

Figure E2. Directed acyclic graph for the hypothesized relationship between maternal levels of human milk oligosaccharides and clinical infections in early childhood. Dotted lines represent where a relationship has not been demonstrated empirically.
